# Gallbladder-preserving cholelithotomy in selected patients with symptomatic cholelithiasis: A case series

**DOI:** 10.1016/j.ijscr.2025.112097

**Published:** 2025-10-25

**Authors:** Hakan Özgür Akıncı, Ozan Şen, Rıfat Tokyay, Deniz Dalayman, Mehmet Tekinel

**Affiliations:** a General Surgery Department, Acibadem Fulya Hospital affiliated with Acibadem University, Istanbul, Turkey; b Nişantaşı University Medical Faculty Department of General Surgery, Turkey; c General Surgery Department, Surgimed Clinic Fulya, Sisli, Istanbul, Turkey; d Radiology Department, Acibadem Fulya Hospital, Sisli, Istanbul, Turkey

**Keywords:** Cholelithiasis, Gallbladder preserving surgery, Cholelithotomy, Gallstone removal, Quality of life, Case series

## Abstract

**İntroduction and importance:**

Laparoscopic cholecystectomy (LC) is the gold standard for the treatment of symptomatic cholelithiasis, but is associated with post cholecystectomy problems in some patients. However, some patients refuse cholecystectomy against organ loss. In suitable patients we employed gallbladder preserving cholelithotomy (GPC) with mini-incision to rid of their stones. Our aim is to introduce our technique of GPC and investigate its safety and efficacy.

**Case presentation:**

Fifty-five biliary colic patients with a strong desire to retain their gallbladders harboring ≤3 gallstones with diameters <3 cm, wall thickness ≤3 mm, and gallbladder ejection fractions >50 % underwent GPC. Assessment of the gallbladder features by ultrasonography every 3 months in the first postoperative year and annually thereafter. We conducted the gastrointestinal quality of life index (GIQLI) survey in the 6th postoperative month.

**Clinical discussion:**

Fifty-two patients had their gallstones successfully removed. We did not encounter any perioperative complication. We converted to laparoscopic cholecystectomy in three patients, one with multiple polyps and two with inflamed mucosa. Gallbladder ejection fractions in the 3rd postoperative month were statistically better than the preoperative period. The patients with preoperative symptoms were symptom free. The median follow-up time was 3 years. There was no stone recurrence.

**Conclusion:**

GPC is a simple, safe and acceptable procedure. It may be employed in suitable symptomatic patients.

## Highlights

• Gallbladder preserving cholelithotomy may be an alternative option in selected patients with cholelithiasis who do not want their gallbladders removed.
• A functional gallbladder is essential in gallbladder preserving cholelithotomy to minimize stone recurrence.
• Stone recurrence and other complications are rare if only suitable patients are selected for cholelithotomy

## 1 Introduction

Dr. Erich Mühe performed the first laparoscopic cholecystectomy (LC) in 1985. Thereafter, LC became the gold standard for treating cholelithiasis [1]. Although LC is generally, considered safe, intraoperative and early postoperative complications can occur in 1–3 % of the cases [2]. Furthermore, patients may later develop post cholecystectomy symptoms such as diarrhea, bloating, nausea, vomiting, or abdominal pain [[2], [3], [4]].

Chinese people, due to their traditional cultural beliefs, are against organ loss and wish to protect their gallbladders even if they have symptomatic gallstones. Therefore, laparoscopic gallbladder-preserving cholelithotomy (GPC), defined as the removal of gallstones without removing the gallbladder, has been introduced and performed with improved outcomes by surgeons in China [5]. Western pediatric surgeons have done GPCs in children with hereditary spherocytosis and gallstones as well as children with symptomatic cholelithiasis [6,7]. GPC differs from previously attempted percutaneous cholelithotomy and drainage in that, after stone removal, the fundus incision is closed primarily without any drains [[8], [9], [10], [11]].

Some patients treated at our clinic were also keen on the preservation of their gallbladders. Among them, we carefully evaluated those suitable for GPC and performed GPC with mini-incision on 52 of them. In this study, we aimed to describe our technique and present our six and a half years of experience with GPC. This is the first study about GPC in Turkey investigating whether GPC is a reliable and admissible option in patients who don't want to lose their gallbladder.

## 2 Methods

### 2.1 Preoperative evaluation and patient selection

In this study, data from patients who applied to our clinic due to symptomatic cholelithiasis between January 2018 and January 2024 were used. Patient inclusion criteria were strong desire for the preservation of their gallbladders, a history of biliary colic, ultrasonography showing ≤3 gallstones with diameters <3 cm, gallbladder wall thickness ≤ 3 mm, and postprandial gallbladder ejection fraction >50 %. Biliary colic was defined which symptoms include severe pain attacks within 30–60 min after eating fatty meals and typically appeared in the epigastrium or right upper quadrant of the abdomen, lasting more than 15 to 30 min, pain radiating to the back and the positive effect of analgesics [12].

We excluded patients with asymptomatic cholelithiasis, acute cholecystitis or cholangitis, suspicion of malignancy, common bile duct stones, biliary pancreatitis, pregnancy, body mass index (BMI) > 29 kg/m2, previous upper abdominal surgery or endoscopic sphincterotomy. Gastroscopy and routine blood tests were performed on each patient before surgery. Patients who were found to have gastritis or peptic ulcer on endoscopy and whose symptoms resolved with medical therapy were excluded from the study. We measured gallbladder ejection fractions by abdominal ultrasonography performed during fasting and 90 min after a fatty meal. The long axis and the transverse sections of the gallbladder were measured by the ellipsoid method. Gallbladder volume was calculated as follows: Gallbladder volume (ml) = 0.52 x length x width x height [13]. A >50 % postprandial reduction of gallbladder volume was considered sufficient for good function. The same radiologist made all the measurements. This study has been reported in line with PROCESS criteria [32].

### 2.2 Ethical approval and informed consent

Our study was conducted in line with Declaration of Helsinki and ethical approval was obtained. All participating patients gave written informed consent.

### 2.3 Case presentation

**Fig. 1:**
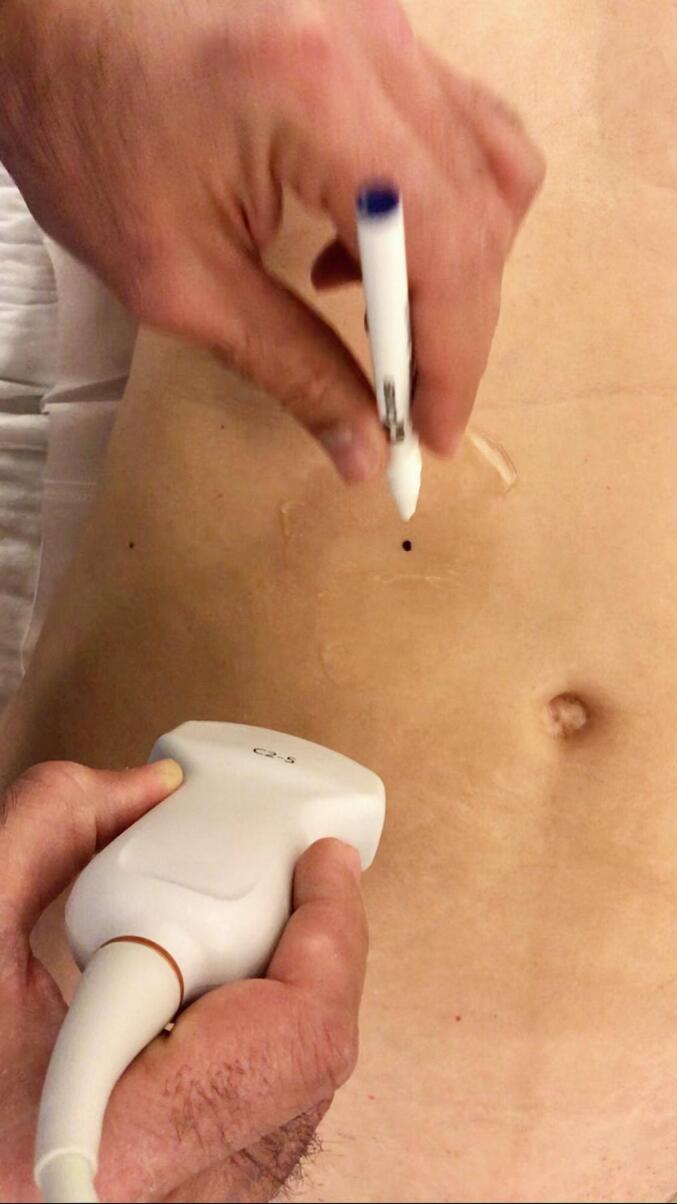
Locate the incision site with ultrasonography.

**Fig. 2:**
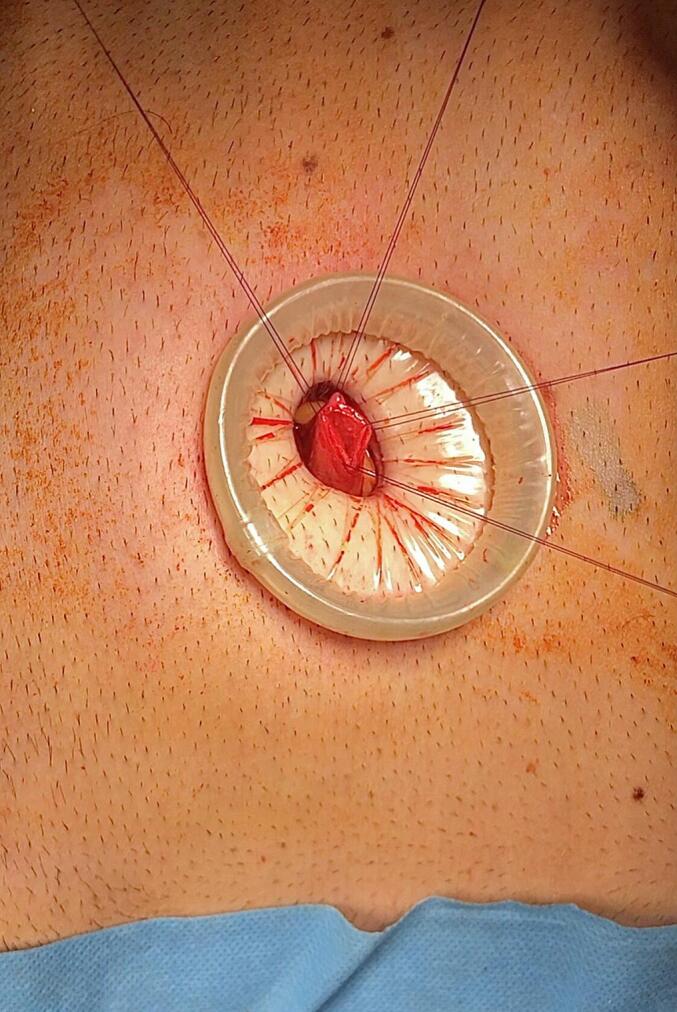
The fundus of the gallbladder was pulled up to the wound and incised.

**Fig. 3:**
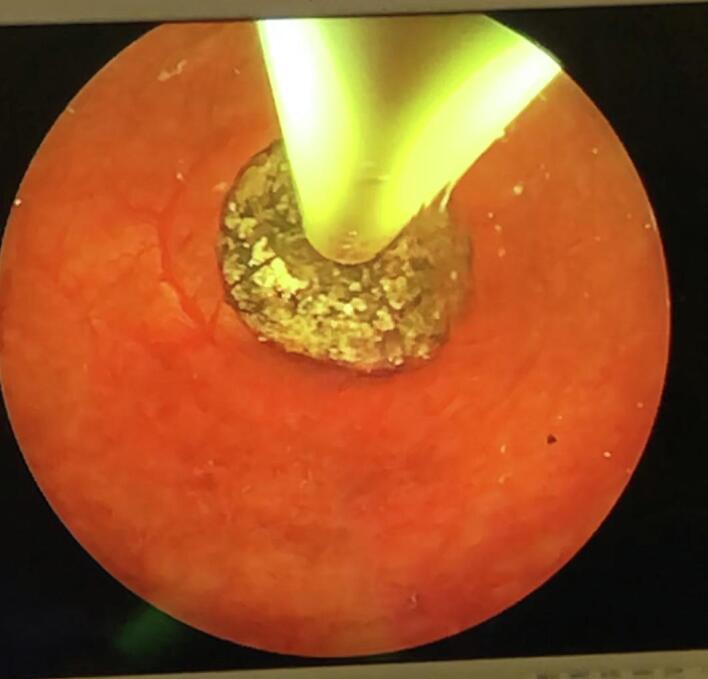
The gallstones were removed with an extractor.

**Fig. 4:**
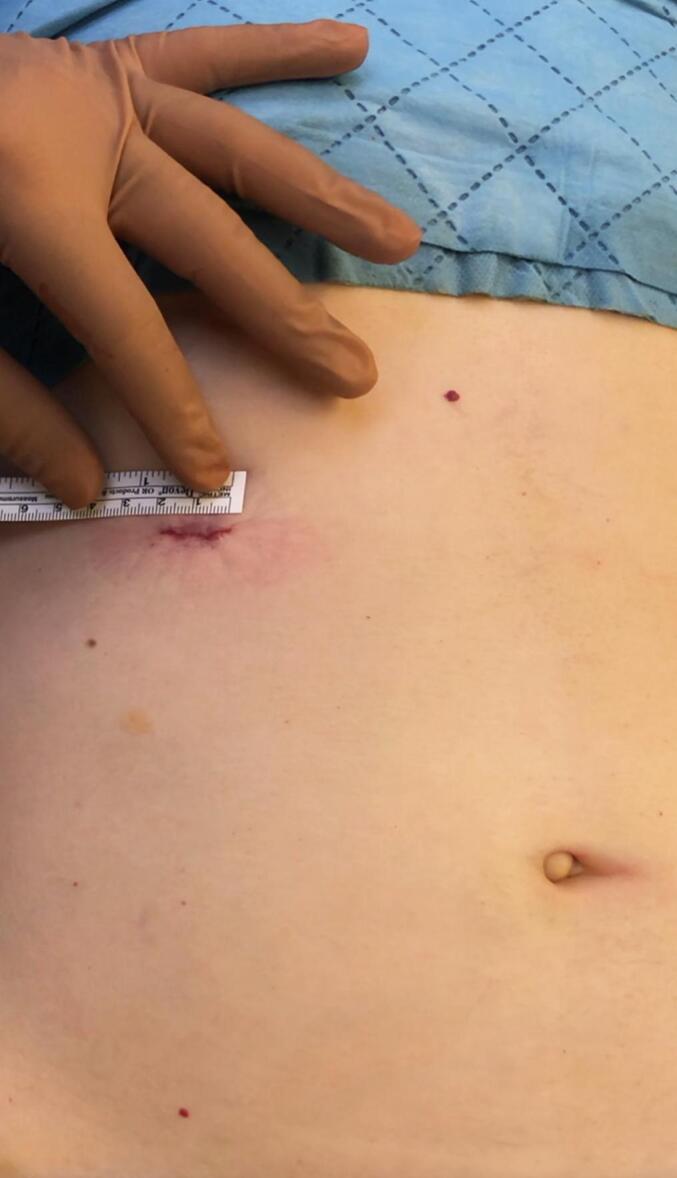
Right subcostal incision.

Under general anesthesia, we adjusted the operating table to put the gallbladder in the subcostal region and to locate the incision site nearest to the abdominal wall utilizing ultrasonography (Fig. 1). A right subcostal mini-laparotomy (1.5–2.5 cm) was performed, and a wound protector (Alexis®wound protector, Size: XS, Applied Medical) was inserted. The fundus of the gallbladder was pulled up to the wound and incised (Fig. 2). A 10 mm rigid nephroscope (Karl Storz®) was inserted, bile was aspirated, and the gallbladder filled with saline. The gallstones were removed with an extractor (Perc NCircle® Nitinol Tipless Stone Extractor, Cook Medical) or a lithotripsy basket (Fig. 3). Before closing the fundal incision, the gallbladder was checked with the endoscope for any bleeding or residual stones. The patency of the cystic duct was confirmed by observing bile reflux from the common bile duct. The fundal incision was closed with double layer running absorbable monofilament sutures (6/0 Monosyn® Braun for mucosa and 5/0 PDS® Ethicon for serosa). The right subcostal incision was closed without a drain (Fig. 4).

### 2.4 Postoperative follow-up

Patients were evaluated clinically and by ultrasound every three months for the first year and annually thereafter. Only Ursodeoxycholic acid (UDCA, 10 mg/kg/d) was recommended to all patients for 3 months. Gallbladder ejection fractions were evaluated by ultrasonography at the 3rd month after surgery by the same radiologist. We prospectively planned to evaluate the patient satisfaction by applying the gastrointestinal quality of life index (GIQLI) questionnaire was conducted with e-mail at the end of the 6th postoperative months to all patients. GIQLI is a validated tool for measuring the quality of life in patients with gastrointestinal diseases. It consists of 36 multidimensional items, including symptoms and physical, emotional, and social dysfunction related to gastrointestinal diseases or their treatments. Each answer is scored from 0 to 4 points. The total GIQLI score is the sum of all item scores. The higher the total score, the better the quality of life [14].

### 2.5 Statistical analysis

Mean, standard deviation, median, frequency and ratio values were used in the descriptive statistics of the data. The status of quantitative variables was measured by Kolmogorov-Smirnov and Shapiro-Wilk tests. In the analysis of dependent continuous variables showing normal distribution, Paired samples *t*-test was used. SPSS 26.0 (IBM Corp. Released 2019. IBM SPSS Statistics for Windows, Version 26.0. Armonk, NY: IBM Corp) and GPower [15] were used in the analyses. In the conducted post-hoc analysis, it was seen that the study reached sufficient power (d: 1.408, act power: 1).

## 3 Results

A total of 645 patients with symptomatic cholelithiasis applied to our clinic. Seventy patients met the inclusion criteria (11 %). Fifty-five patients (35 females, 20 males) who were initially planned for GPC were included in this study. These patients prefer GPC to preserve their gall bladders and to avoid the potential adverse effects associated with post-cholecystectomy syndrome. 15 patients did not proceed with the operation and were not included in this study. We performed cholecystectomy in 3 patients in whom we detected mucosal inflammation and multiple polyps that could not be detected on ultrasonography during intraoperative endoscopic gallbladder evaluation. GPC was successfully performed in 52 patients. The mean age was 45.8 ± 11.1 years (16–66 years). The mean operation time was 56.5 ± 15.1 min. Cholesterol polyps were also detected in 3 patients during GPC and polypectomy was performed. The pathology report indicated a cholesterol polyp in these patients. Estimated blood loss was 10 ml. The patient characteristics and the timelines of the procedure are shown in Table 1.

**Table 1:** Patient's characteristics and timelines.

Number (n)	55
Age (y)^a^	45.8 ± 11.1
Sex (M/F)	20/35
BMI (kg/m2)^a^	26.1 ± 2.0
Gallstones size (mm)^a^	12.2 ± 6.2
Gallstone number (n) single/2/3	35/15/5
Operation time(min)^a^	56.5 ± 15.1
Hospital stay (h)^a^	14.8 ± 4.7
Stone recurrence n (%)	1 (1.9 %)

BMI: body mass index.

a Values are mean ± standard deviations.

**Fig. 5:**
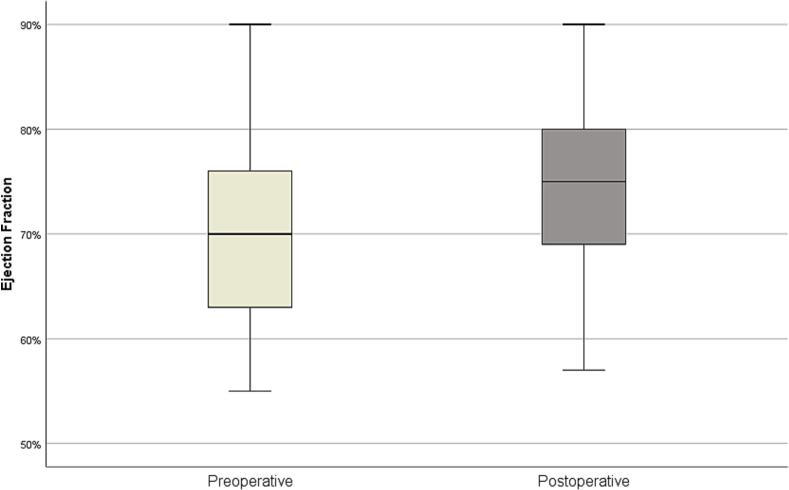
Comparing preoperative and postoperative gallbladder ejection fractions.

No perioperative complications (bile leakage, cholecystitis, or wound problems) were encountered in any of the patients. All patients were followed up for a median of 3 years (range: 9–78 months). Postoperative initial ultrasonography detected a missed gallstone in one patient (2.3 1.8 %). This patient refused cholecystectomy and is currently asymptomatic at 4 years post GPC. When comparing preoperative and postoperative ejection fractions, an average increase of 5.37 % was observed in postoperative measurements (70.20 ± 8.53 vs. 75.00 ± 8.14, *p* < 0.001) (Fig. 5).

GIQLI mean scores obtained from the responses to the questionnaires conducted during the 6-month postoperative follow-up showed patient satisfaction in all items (Table 2) and subgroups (Table 3).

**Table 2:** GIQLI items score.

Item	Score (mean ± SD)
Bowel urgency	3.82 ± 0.39
Diarrhea	3.88 ± 0.33
Heartburn	4.00 ± 0.00
Disturbing bowel movements	3.82 ± 0.39
Enjoyment in eating	3.76 ± 0.44
Restricted eating	3.76 ± 0.44
Coping with stress	3.53 ± 0.62
Bowel frequency	3.8 ± 0.41
Regurgitation	3.95 ± 0.23
Slow eating	4.00 ± 0.00
Fatigue or exhaustion	3.60 ± 0.50
Life satisfaction	3.59 ± 0.62
Abdominal pain	3.76 ± 0.75
Epigastric fullness	3.65 ± 0.49
Gas and bloating	3.24 ± 0.66
Flatus	3.29 ± 0.47
Burping and belching	3.65 ± 0.49
Abdominal noises	3.53 ± 0.51
Sadness	3.82 ± 0.39
Nervousness	3.71 ± 0.47
Frustration about illness	3.76 ± 0.40
Feeling unwell	3.47 ± 0.60
Waking up at night	3.4 ± 0.50
Physical change	3.82 ± 0.39
Physical strength change	3.76 ± 0.44
Loss of stamina	3.82 ± 0.39
Feeling unfit	3.65 ± 0.49
Daily activities	3.82 ± 0.39
Leisure activities	3.35 ± 0.49
Feeling restricted	4.00 ± 0.00
Personal relations	3.94 ± 0.24
Sexual life	3.82 ± 0.39
Dysphagia	4.00 ± 0.00
Constipation	3.59 ± 0.51
Nausea	3.94 ± 0.24
Blood in stool	4.00 ± 0.00

GIQLI: gastrointestinal quality of life index.

SD: standard deviation.

**Table 3:** GIQLI total and subgroup scores.

Group	Score (mean ± SD)
GIQLI Total	135.7 ± 7.7
Core symptoms	33.0 ± 2.92
Psychological items	18.7 ± 1.9
Physical items	25.9 ± 2.2
Social items	19.1 ± 0.9
Disease-specific items	39.2 ± 1.2

GIQLI: gastrointestinal quality of life index.

SD: standard deviation.

## 4 Discussion

Current literature emphasizes that storage and postprandial secretion of bile is essential for the digestive system's circadian rhythm, enterohepatic circulation of bile acids, and normal metabolism. The continuous flow of bile to the duodenum following cholecystectomy may enhance duodenal bile reflux, alkaline gastritis, and gastroesophageal reflux, causing dysplasia [[16], [17], [18]]. GPC is frequently performed in China in patients with cholelithiasis and functional gallbladder to avoid the undesirable effects of cholecystectomy [5,[8], [9], [10], [11]]. We emphasized to our patients with symptomatic cholelithiasis that cholecystectomy is currently the widely accepted standard to prevent complications and recurrence of cholelithiasis, and GPC surgery is not considered as the first choice. However, not only the Chinese, but also some patients from our country and abroad insist on protecting their gallbladders. These patients desire this method due to their cultural beliefs or internet and social media research. Some of our colleagues in our country, who are aware of the early and late complications of cholecystectomy, also refer their patients for the GPC procedure.

### 4.1 Selection of suitable patients

In our study, we considered biliary colic as symptomatic gallstone disease. Biliary colic due to gallstone in the gallbladder as an indication of operation is somewhat controversial. Some authors recommend observation without intervention [12]. An intervention may be justified before serious complications require riskier operations, such as not postponing hernia repair until intestinal obstruction occurs. Moreover, our GPC technique is a relatively simple procedure like hernia repair and the risk of life-threatening complications is almost none.

Our inclusion criteria (strong desire for the preservation of their gallbladders, a history of biliary colic, ultrasonography showing ≤3 gallstones with diameters <3 cm, gallbladder wall thickness ≤ 3 mm, and postprandial gallbladder ejection fraction >50 %) were similar to those reported in the literature [[8], [9], [10], [11]]. As described in the methods section, the GPC candidates underwent a preoperative evaluation of their gallbladder ejection fractions. Recent studies [9] regarded a postprandial contraction of the gallbladder of 30 % as sufficient. However, Pauletzki et al. [19] stated that a volume reduction of <60 % increases the risk of recurrence after GPC. In this study, postprandial ejection fractions above 50 % were considered functional gallbladders suitable for GPC surgery.

### 4.2 Operative procedure and safety

Early attempts at GPC around the end of the 1980s by Kellet et al. [20] and Donald et al. [21] were abandoned due to long hospitalization times and high stone recurrence rates. These authors drained the gallbladder for at least ten days with a Foley's catheter instead of closing the fundus primarily after stone removal. Chinese surgeons did not use gallbladder drainage, but they usually performed laparoscopic GPC through several ports with abdominal insufflation [[8], [9], [10], [11]]. Our technique differs from GPC described in current Chinese studies. We didn't use a laparoscopic approach for GPC, and we utilize preoperative, intraoperative, and postoperative ultrasonography extensively in selecting patients suitable for GPC, determining the site of the single mini-incision (1.5–2.5 cm), and postoperative follow-up. The gallbladder fundus was easily pulled up to the incision in all our patients. We believe perioperative use of ultrasonography in locating the projection of the fundus closest to the abdominal wall is critical. The fundus can then easily be pulled up to the incision, stones removed, and the fundus primarily closed. If the fundus cannot be pulled to the incision adequately, the surgeon can still convert to laparoscopic cholecystolithotomy or cholecystectomy [11].

Bile leakage was also a rare complication of GPC in other Chinese studies [[8], [9], [10], [11]]. On the other hand, postoperative complications of cholecystectomy, although relatively seldom when compared to the number of cholecystectomies done worldwide, can be quite detrimental if encountered. The incidence of bile duct injury and bile leakage in post cholecystectomy patients has been reported between 0.3 % and 1.4 %. Other studies reported injury and leakage rates as high as 1.5–2.4 % [22]. Such serious vascular or bile duct injuries are not encountered in GPC surgery because the hepato-cystic triangle (Calot's) and hepatic hilum are technically untouched [[8], [9], [10], [11]]. The safety of our technique is depicted in the results section. We used double-layer monofilament absorbable sutures for the closure of the fundal incision. No perioperative complications (bile leakage, cholecystitis, or wound problems) were encountered in any of the patients. One other contribution of gallbladder-preserving surgery to the medical literature is that it is safe to close the non-inflamed gallbladders primarily. Surgeons previously performed cholecystectomies routinely if accidental lacerations or incidental stones were encountered during laparotomy [23], just as splenectomy was done previously for even minor splenic lacerations [24].

### 4.3 Patient satisfaction

The GIQLI questionnaire has effectively been used to compare patients who underwent LC versus open cholecystectomy or single incision LC [25,26]. Additionally, the questionnaire includes questions regarding complaints of abdominal pain, nausea, vomiting, dyspepsia, distension, diarrhea or stool urgency, and bloating, regarded as symptoms of post-cholecystectomy syndrome [27]. A review analyzing 11 studies stated that flatulence, diarrhea, heartburn, belching, and nausea were the most common symptoms post-cholecystectomy [28]. Post-cholecystectomy syndrome is a factor that reduces patient satisfaction. Prior GIQLI studies after cholecystectomy consistently show clinically meaningful improvements in total scores within months of surgery. Nevertheless, population-based comparisons reveal that, despite good overall quality-of-life, the GI-symptom domain can remain worse than background controls, driven by diarrhea, urgency, bloating and related complaints. Wanjuro et al. reported that GIQLI score assessed four years after cholecystectomy was higher compared to preoperative values, although the gastrointestinal symptom subscore was significantly reduced in subgroup analyses [29]. In our study of gallbladder-preserving cholelithotomy (GPC), we administered the GIQLI at 6 months post-operatively and observed high patient-reported satisfaction across all items and subgroups (Table 2, Table 3). We think preserved gallbladder function may explain why selected patients experience favorable symptom control after GPC. We report absolute post-operative GIQLI values and acknowledge that preoperative GIQLI was not collected. Additional diagnosis and treatment methods may be required in 10–40 % of patients to rule out pathologies other than post-cholecystectomy syndrome [[2], [3], [4]]. The gallbladder functions of our post-GPC patients were significantly better than before surgery and similar to those of the recent literature confirming that a functional gallbladder left in situ largely eliminates the risk of post-cholecystectomy syndrome [30]. This may be the reason for our high patient satisfaction rates.

### 4.4 Gallstone recurrence

The major drawback of GPC is, of course, the possibility of gallstone recurrence. We detected a gallstone on initial routine ultrasonography in one patient after GPC. It was probably a missed stone. In a recent meta-analysis evaluating 14 randomized controlled trials involving 2030 GPC patients, the recurrence rate of gallstones was 10.11 % after more than 15 years of follow-up [31]. Liu et al. reported their 1-year cumulative gallstone recurrence rate of GPC as 0.83 %. The rate increased proportionally with the postoperative time, reaching 7.94 % during the 14-year follow-up period, but was unchanged between 14 and 23 years [8]. Qu et al. demonstrated similar results in 216 patients who underwent GPC [10]. They suggested the indication for GPC should be limited to patients with one or a small number of stones (≤ 3) to decrease stone recurrence. We followed similar strategies described in these studies. Our recurrence rate, albeit the follow up period is shorter, is similar to these studies. Although the effects of ursodeoxycholic acid on dissolution of gallbladder stones is questionable it may prevent new gallstone formation [10]. Therefore, we recommended to all our patients the postoperative use of ursodeoxycholic acid for 3 months.

Our study's one limitation was its small number of cases. The main reason for this relatively small number of GPC cases over 6 years was our strict exclusion criteria. During the study period we agreed to perform GPC only on 52 of 645 symptomatic gallstone patients (8 %).

Nevertheless, the strengths of our study include it being the first ultrasound-assisted GPC study and the first to assess the GIQLI scores post-GPC. Besides, it is one of the rare articles about GPC outside of China. Therefore, we think that our article will contribute to the scientific literature.

## 5 Conclusions

GPC may be a safe, effective, and admissible treatment option in suitable symptomatic patients with gallstones who ardently desire to have their gallbladders left in situ. Biliary colic patients who deny cholecystectomy and do not want to go through prolonged dietary restrictions should not be deprived of their right to get rid of the symptoms and the risk of complications of their gallstones, even if gallstone recurrence may still be a potential problem which can be reduced with careful patient selection. GPC, which has a very low complication rate, should be researched and developed through randomized and large-scale studies.

## CRediT authorship contribution statement

Each author have participated sufficiently in the work to take public responsibility for appropriate portions of the content. All authors met all of the following criteria:

- Substantial contributions to the conception or design of the work; or the acquisition, analysis, or interpretation of data for the work; O.H.A., O.Ş., D.D., R.F. and M.T.
- Drafting the work or revising it critically for important intellectual content; O.H.A., O.Ş., D.D. and M.T.
- Final approval of the version to be published; O.H.A., O.Ş., D.D. and M.T.
- Agreement to be accountable for all aspects of the work in ensuring that questions related to the accuracy or integrity of any part of the work are appropriately investigated and resolved.

O.H.A operated the patients.

O.H.A and O.Ş wrote the first draft of the manuscript.

O.H.A and O.Ş wrote the final draft of the manuscript.

O.H.A, O.Ş and M.T made the corrections in English. All authors have read and approved the final report.

## Consent

Written informed consent was obtained from all the participants. A copy of the written consent is available for review by the Editor-in Chief of this journal on request.

## Ethical approval

Our study was conducted in line with Declaration of Helsinki and ethical approval was obtained.

## Guarantor

Ozan Sen.

## Funding

This research has not received any funding to be conducted.

## Declaration of competing interest

None.
